# mRNA-Seq and MicroRNA-Seq Whole-Transcriptome Analyses of Rhesus Monkey Embryonic Stem Cell Neural Differentiation Revealed the Potential Regulators of Rosette Neural Stem Cells

**DOI:** 10.1093/dnares/dsu019

**Published:** 2014-06-17

**Authors:** Yuqi Zhao, Shuang Ji, Jinkai Wang, Jingfei Huang, Ping Zheng

**Affiliations:** 1 State Key Laboratory of Genetic Resources and Evolution, Kunming Institute of Zoology, Chinese Academy of Sciences, 32 East Jiaochang Road, Kunming, Yunnan 650223, China; 2 Yunnan Key Laboratory of Animal Reproductive Biology, Kunming Institute of Zoology, Chinese Academy of Sciences, 32 East Jiaochang Road, Kunming, Yunnan 650223, China; 3 University of Chinese Academy of Sciences, Beijing, China; 4 Department of Microbiology, Immunology and Molecular Genetics, University of California, Los Angeles, Los Angeles, CA 90095, USA; 5 Kunming Institute of Zoology, Chinese University of Hongkong Joint Research Center for Bio-resources and Human Disease Mechanisms, Kunming, Yunnan 650223, China

**Keywords:** rhesus monkeys, embryonic stem cells, neural differentiation, transcriptome, microRNAomes

## Abstract

Rosette neural stem cells (R-NSCs) represent early stage of neural development and possess full neural differentiation and regionalization capacities. R-NSCs are considered as stem cells of neural lineage and have important implications in the study of neurogenesis and cell replacement therapy. However, the molecules regulating their functional properties remain largely unknown. Rhesus monkey is an ideal model to study human neural degenerative diseases and plays intermediate translational roles as therapeutic strategies evolved from rodent systems to human clinical applications. In this study, we derived R-NSCs from rhesus monkey embryonic stem cells (ESCs) and systematically investigated the unique expressions of mRNAs, microRNAs (miRNAs), and signalling pathways by genome-wide comparison of the mRNA and miRNA profilings of ESCs, R-NSCs at early (R-NSCP1) and late (R-NSCP6) passages, and neural progenitor cells. Apart from the R-NSCP1-specific protein-coding genes and miRNAs, we identified several pathways including Hedgehog and Wnt highly activated in R-NSCP1. The possible regulatory interactions among the miRNAs, protein-coding genes, and signalling pathways were proposed. Besides, many genes with alternative splicing switch were identified at R-NSCP1. These data provided valuable resource to understand the regulation of early neurogenesis and to better manipulate the R-NSCs for cell replacement therapy.

## 1. Introduction

Neurogenesis takes place robustly during the development of fetal nervous system and continues postnatally and in adults.^1,2^ It relies on multipotent neural stem cells (NSCs), which can generate different cell types of nervous system. Based on their localization and developmental stages, NSCs exhibit distinct spatial and temporary properties in terms of the neural specification and differentiation potentials.^3^ NSCs can also be derived from pluripotent stem cell (PSC) *in vitro* neural differentiation.^4^ Different protocols have been developed to derive and propagate NSCs from PSCs.^5,6^ Similar to their *in vivo* counterparts, PSC-derived NSCs showed distinct neural differentiation competence according to the protocols utilized. Currently, PSC *in vitro* neural differentiation is widely employed to study the regulation of neurogenesis and to generate NSCs or neurons in cell replacement therapy of neurodegenerative and neuropsychiatric disorders.^7,8^ However, most of the PSC-derived NSCs were lack of *bona fide* NSC properties, i.e. they displayed restricted neural differentiation fate and failed to generate all cell types of neural lineage, or they lost the ability to respond to regionalization cue.^3^ These shortcomings hampered their potential applications in basic research as well as in cell replacement therapy. Recently, Elkabetz *et al.*^9^ identified an early stage of NSC population during the process of human embryonic stem cell (ESC) neural differentiation. These earlier NSCs exhibited the unique rosette morphology and were named as rosette NSC (R-NSC). Compared with other NSCs ever described, R-NSC possesses true NSC properties (i.e. broad neural differentiation potential and regionalization ability) and is considered as stem cells of neural lineage.^9^ Albeit with high potential in the study of neurogenesis as well as cell replacement therapy, R-NSC is difficult to be maintained and propagated in culture. Moreover, whether R-NSC exhibits a high risk of tumourigenicity following transplantation remains elusive. In these regards, an intensive study of R-NSC is required in order to thoroughly understand the molecular mechanisms regulating the self-renewal and multipotency of the R-NSCs. However, few works were carried out and little information was obtained regarding the molecular properties of R-NSC in human and even in mice. To our knowledge, only one study examined the human R-NSC-specific genes by microarray analysis.^9^

Rhesus monkey (*Macaca mulatta*) has >90% (92.5–95%) DNA homologue to human and has long been considered as a reliable non-human primate model to study various human diseases and to assess the preclinical safety of medical treatments.^10,11^ In particular, the cell replacement therapy based on the invention of induced PSC (iPSC) technology called for safety assessment in primates before its application in clinics.^12,13^ Thus, rhesus monkey plays intermediate translational roles as therapeutic strategies evolve from rodent systems to human clinical applications. Rhesus monkey ESCs (rESCs) and iPSCs have successfully been derived in several laboratories around the world, and they shared a number of properties with human ESCs.^14–16^ Furthermore, rhesus monkey animal models for neural degenerative diseases including Huntington's disease,^17^ Parkinson's disease,^18^ and spinal cord injury^19^ were demonstrated. Investigating the rESC-derived R-NSCs and transplanting them into the monkey disease models would be a perfect strategy to evaluate the cell replacement therapy in human neural degenerative diseases.

To understand the molecular characters and especially the molecular interaction networks implicated in the maintenance of unique functional properties of R-NSC in rhesus monkey, we performed *in vitro* neural differentiation of rESCs according to the protocol developed by Elkabetz *et al.*^9^ Using the mRNA and microRNA (miRNA) deep sequencing-based systemic analysis, we investigated the whole-genome mRNA and miRNA profilings of the ESCs, R-NSCs at early and late passages (R-NSCP1 and R-NSCP6, respectively), and neural progenitor cells (NPCs) which completely lost the characteristics of R-NSCs. Our results described the dynamics of transcriptomes and miRNAomes during ESC neural differentiation. Comparisons among the four differentiation stages uncovered a hand of R-NSCP1-specific or prevalent mRNAs, miRNAs, and signalling pathways. The regulatory interactions of these molecules were also proposed.

## 2. Materials and methods

### 2.1. rESC culture and induction of neural differentiation

rESCs (line IVF3.2) were cultured on feeder cells of mitotically inactivated mouse embryonic fibroblasts as described previously.^20^ Embryoid body (EB) differentiation was employed to induce neural differentiation. Briefly, rESC colonies were transferred onto tissue culture dishes coated with 1% agar and differentiated in ESC culture medium depleted of basic fibroblast growth factor (bFGF) to induce EB differentiation. EBs were then transferred onto extracellular matrix-coated (10 μg/cm^2^, Sigma-Aldrich) tissue culture dishes to spread and propagate in DMEM/F12 medium (Gibco) supplied with ITS (Sigma-Aldrich), 2.5 µg/ml of fibronectin (Millipore), 60 µM putrescine (Sigma-Aldrich), and 20 ng/ml of bFGF. Neural tube-like structures could be observed in EB outgrowth, and rosette-like clusters were then manually separated and expanded according to the protocol described by Elkabetz *et al.*^9^ with minor modifications. In brief, rosette clusters were replaced on culture dishes pre-coated with 15 µg/ml of polyornithine plus 1 µg/ml of laminin (Po/Lam) (Sigma-Aldrich) in N2 medium supplemented with SHH (200 ng/ml; R&D), FGF8 (100 ng/ml; Sigma-Aldrich), ascorbic acid (0.2 mM; Sigma-Aldrich), and brain-derived neurotrophic factor (BDNF, 20 ng/ml; R&D). The first passage of rosette culture was defined as R-NSCP1. To propagate the R-NSCs, only rosette structures were picked and passed at the density of 100–400 × 10^3^ cells/cm^2^ when the growth reached 80% confluence. R-NSCs after the first passage were propagated in Po/Lam culture dishes in N2 medium with 0.2 mM ascorbic acid, 20 ng/ml of BDNF, 500 ng/ml of SHH, 500 ng/ml of Dll4 (R&D), and 500 ng/ml of Jagged-1 (R&D). Under this culture condition, rosette structure can be maintained for up to five generations. At the sixth generation, the rosette structure was no longer visible and the cells were defined as R-NSCP6. NPCs were derived from R-NSCs with extended culture on Po/Lam culture dishes in N2 medium with 0.2 mM ascorbic acid, 20 ng/ml of BDNF, 20 ng/ml of FGF2, and 20 ng/ml of EGF for >100 days.^21^ The differentiation efficiency from R-NSCs to NPCs under this condition was close to 100% based on cell morphology as well as calculating the percentages of cells labelled with stage-specific marker S100b.

### 2.2. Patterning and neural differentiation of R-NSCs and NPCs

R-NSCs were examined for neural regional patterning potentials.^9^ The following factors were used to coax the regional patterning of R-NSCs: 200 ng/ml of SHH plus 1 µM retinoic acid (RA) for posterior patterning, 200 ng/ml of SHH plus 100 ng/ml of FGF8 for anterior patterning, 40 ng/ml of Wnt3a (R&D) for dorsal patterning, and 200 ng/ml of SHH for ventral patterning.

NPCs were examined for their neural differentiation abilities. For neuronal differentiation, NPCs were replated onto Po/Lam-coated plates at a density of 2.5 × 10^4^ cells/cm^2^ in DMEM/F12 medium supplemented with N2, B27 (Gibco), BDNF (20 ng/ml), glial derived neurotrophic factor (20 ng/ml), 1 mM dibutyryl cyclic AMP (Sigma-Aldrich), and ascorbic acid (200 nM). For astrocyte and oligodendrocyte differentiation, 1% fetal calf serum (Gibco) was added in the above media.^22,23^

### 2.3. Immunofluorescent staining and quantitative reverse transcription–polymerase chain reaction

For immunofluorescent staining, cells were fixed with 4% paraformaldehyde in PBS for 20 min at room temperature followed by permeabilization in 0.2% Triton X-100 in PBS for 10 min. After blocking with 3% bovine serum albumin, cells were incubated with primary antibodies (Supplementary Table S1) overnight at 4°C. Cells were then rinsed in PBS (3× 5 min) followed by incubation with fluorescein isothiocyanate-conjugated or Texas red-conjugated secondary antibody for 1 h. Negative controls were carried out without the addition of the primary antibody.

Cell samples used for cDNA preparation and quantitative reverse transcription–polymerase chain reaction (qRT–PCR) validation were different from those used for RNA-Seq. Total RNA was extracted using Trizol LS Reagent (Invitrogen) according to the manufacture's instruction. Potential contamination from genomic DNA was eliminated by DNase digestion. RNA was reverse-transcribed into single-strand cDNA. Aliquots of cDNA were used as templates for qRT–PCR. The PCR consisted of 1 µl of 1 : 3-diluted cDNA, 10 µl of SYBR green-*Taq* mixed solution (Sigma), and 20 pmol each of 5′ and 3′ primers (Supplementary Table S2) in a total volume of 20 µl and was performed in an Opticon thermal cycler (Bio-Rad) for 35 cycles with denaturation at 95°C for 15 s, annealing at 55–58°C for 30 s, and extension at 72°C for 15 s. RNA without reverse transcriptase treatment was used as negative control.

### 2.4. Sample collection and preparation of the libraries for mRNA and miRNA deep sequencing

In R-NSCP1 culture, a fraction of cells lost the rosette morphology indicative of differentiation. In order to avoid the contamination of R-NSCP1 sample by other cells, only rosette structures were manually collected (R-NSCP1) for downstream RNA extraction. Similarly, pure rosette structures without contamination of differentiated cells were used for R-NSC passage. At passage 6, the rosette structure was no longer formed and all cells in the culture dish were collected (R-NSCP6) for RNA extraction. To get the enough amount of cells for RNA-Seq, ESC neural differentiation was repeated many times and the R-NSCs as well as NPCs from each repeat were pooled for RNA extraction. Therefore, the sample of each stage is the pool of many repeats. mRNA sequencing using HiSeq 2000 was performed at Macrogen Inc. According to the manufacturer's instructions (Illumina), mRNA was purified using poly-T oligo-attached magnetic beads. Following purification, the mRNA was fragmented into small pieces. The cleaved RNA fragments were converted to the first-strand cDNA followed by generation of double-strand cDNAs. These cDNA fragments went through end repair course by adding a single ‘A’ base as well as the adapter ligation. The cDNA libraries prepared from samples of ESCs, R-NSCP1, R-NSCP6, and NPCs were used for paired-end mRNA sequencing by Illumina HiSeq 2000.

For miRNA sequencing, total RNA was ligated to a pair of adaptors at the 5′ and 3′ ends according to the manufacturer's instructions (Illumina). RNA molecules were converted to cDNA and amplified by RT-PCR using adaptor primers. After amplification, library of small RNAs with size of ∼140 bp was purified and used for cluster generation and single-end small RNA sequencing by Illumina HiSeq 2000.

### 2.5. Read mapping and abundance estimate for genes and miRNAs

RNA-seq reads were mapped to the *M. mulatta* genome (MMUL_1.0) using TopHat^24^ (v. 2.0.6) with default settings. Cufflinks^25^ (v. 2.0.2) were used to estimate fragments per kilobase of exon per million fragments mapped (FPKM) values for University of California Santa Cruz Genome Database known genes. According to the previous report that a gene with three FPKMs corresponds to about one transcript in one cell,^26^ we consider the genes with an FPKM value of ≥3 as expressed.

Using the Bowtie software^27^ (v. 2.0.2), miRNA sequence reads were aligned to the rhesus annotated miRNAs in the miRBASE database (Release 19, http://www.mirbase.org/) and the frequency of reads that mapped uniquely to each miRNA was calculated. The adaptor sequences were trimmed from individual reads using a customized Perl script. Reads in which the adaptor sequences were either mutated or absent were discarded. Unmatched sequences were collapsed to obtain a set of unique reads. The reads with sequence length between 15 and 30 were analysed by miRDeep algorithm.^28^ Reads that aligned to more than five positions in the human genome were excluded from the data set, as they may reside in repetitive locus. The potential precursors were excised to generate secondary stem–loop structure using the Vienna RNAfold package (v. 1.8; http://rna.tbi.univie.ac.at).^29^ Finally, miRDeep assigned a score to each read based on: (i) seed sequence homology to known human miRNA, (ii) the presence of a star sequence, (iii) minimum free energy (RNAfold), (iv) energetic stability (Randfold), and (v) frequency of reads that correspond to Dicer processing. A final score was assigned, and the minimum total score by default was one.

### 2.6. Alternative splicing analysis

We conducted the alternative splicing (AS) analysis using the MATS software (version 3.0.7.beta).^30^ Six comparisons were made between any two of the four stages to test if there were 5% or more change of the exon inclusion level (*Ψ*) for exon skipping events, and the events with false discovery rate (FDR) of <0.1 were determined as significant changes between the two stages. The stage-specific AS events were then determined as that with the highest of lowest *Ψ* in a specific stage, and the *Ψ* changes are significant between this stage and any other stages base on MATS calculation. Considering that R-NSCP1 and R-NSCP6 are similar, the AS events did not have to be significant between these two stages when defining AS events as R-NSCP1 or R-NSCP6 specific.

### 2.7. miRNA-target prediction

In order to retrieve information of miRNA targets in rhesus monkeys, we integrated data sets of different sources. First, we retrieved miRNA-target information from the miRNAMap database (version 2.0).^31^ In prediction, the minimum free energy threshold of the miRNA and target duplex was set to −12 kcal/mol and the miRanda score was specified as 120. The predictive parameters of TargetScan and RNAhybrid were set as default values. Secondly, we applied miRDB (http://mirdb.org/miRDB/), which was an online database for miRNA-target prediction and functional annotations based on support vector machines and high-throughput training data sets.^32^ The target score was set to 50 in prediction.

### 2.8. Statistical analysis

Fisher's exact test with Benjamini–Hochberg FDR controls^33^ was performed to identify enriched Gene Ontology (GO) terms and annotated gene sets in the Molecular Signatures Database (MSigDB).^34^ We also used gene expression profiles of many human/mouse tissues from the Genomics Institute of the Novartis Research Foundation (GNF, version 3; http://biogps.org/)^35,36^ to further characterize the transcriptome alterations in ESC neural differentiation.

## 3. Results

### 3.1. Characterization of rESC-derived NSCs

**Figure 1.**
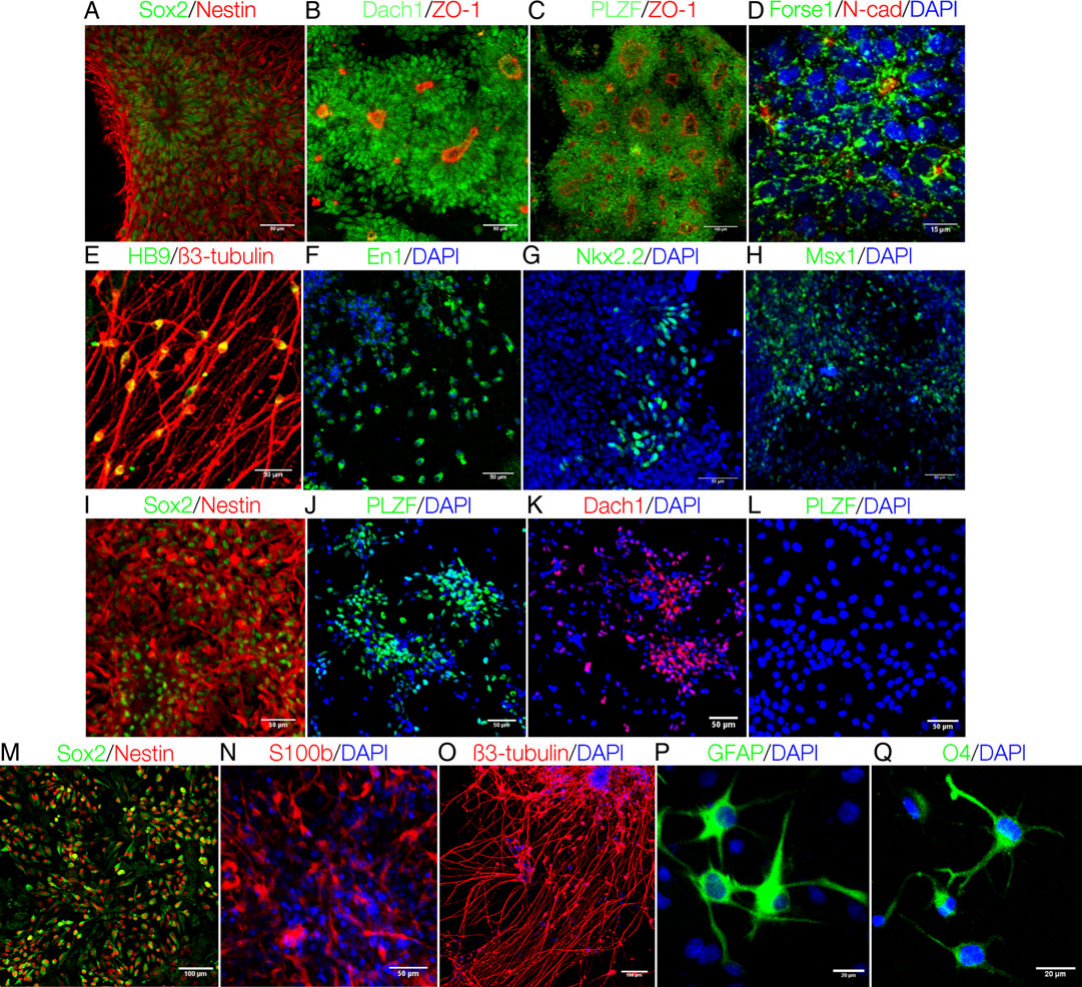
Characterization of rESC-derived NSCs. The common NSC markers Nestin and Sox2 (A); the specific markers Dach1 (B) and PLZF (C), and the polarized distribution of ZO1 in R-NSCP1 (B and C); the co-expression of Forse1 and *N*-cad in R-NSCP1 (D); R-NSCP1 can be coaxed to undergo posterior (E), anterior (F), ventral (G), and dorsal patternings (H). R-NSCP6 cells expressed Sox2 and Nestin (I), PLZF (J), and Dach1 (K). Glial-like NPCs did not express PLZF (L), but expressed Sox2 and Nestin (M), specific marker S100b (N), and can differentiate into neurons (O), astrocytes (P), and oligodendrocytes (Q).

rESCs derived in Dr Wei-zhi Ji's lab were used in this study.^20^ These stem cells have normal karyotype, express ESC markers, and can form three germ layer cells in teratoma formation assay.^20^ According to the neural induction protocol developed by Elkabetz *et al.*,^9^ ESCs at passages 15–30 were sequentially differentiated into early (neural rosette) and late (glial-like) stages of NSCs. The identities of R-NSC and glial-like NSC/NPC were characterized based on the standards described in Elkabetz *et al.*'s study.^9^ Briefly, R-NSCs at passage 1 (R-NSCP1) represented the early stage of NSC and displayed typical morphology, i.e. the columnar epithelial cells radically organized to form rosette structure. They expressed not only the common NSC markers Sox2 and Nestin (Fig. 1A), but also the neural rosette specific markers Dach1 and PLZF (official symbol ZBTB16) (Fig. 1B and C). These early NSCs adopted polarized neuroepithelial structure of anterior central nervous system (CNS) fate and exhibited forebrain precursor cell marker Forse1 (Fig. 1D). They also displayed asymmetric apical localization of tight junction protein ZO-1 and neuroepithelial marker *N*-cadherin (*N*-cad) (Fig. 1B and D). Most importantly, these early R-NSCs possessed broad regional specification competence. For instance, they could be coaxed to differentiate into HB9^+^ spinal motor neurons by SHH/RA posterior patterning cues (Fig. 1E), and into En1^+^ midbrain precursors in response to SHH/FGF8 anterior patterning molecules (Fig. 1F). They also underwent ventral–dorsal specification upon exposure to ventral and dorsal patterning signals such as SHH or Wnt3A, as indicated by the inducible expression of ventral forebrain marker Nkx2.2 (Fig. 1G) and the dorsal marker Msx1 (Fig. 1H).

Although R-NSCP1 exhibits high patterning plasticity and therefore represents the ideal NSC population for a range of biomedical applications, they are difficult to be stably maintained and propagated *in vitro*. In our experimental setting, R-NSCs at the sixth passage (R-NSCP6) lost most of the unique properties described above including typical rosette morphology, the polarized distribution of ZO-1 and *N*-cad, and the responsiveness to patterning cues. However, they retained the partial staining for R-NSCP1 markers PLZF (Fig. 1J) and Dach1 (Fig. 1K), and expressed the common NSC markers Sox2 and Nestin (Fig. 1I). Thus, R-NSCP6 represented a transitional stage between R-NSCP1 and the glial-like NSC/NPC, which completely lost the expression of R-NSC-specific marker PLZF (Fig. 1L) and could be maintained in the presence of FGF2/EGF for long time. These FGF2/EGF-dependent NPCs expressed common NSC markers Nestin and Sox2 (Fig. 1M) as well as stage-specific marker S100b (Fig. 1N). Their NSC identity was further confirmed by the ability to differentiate into neurons, astrocytes, and oligodendrocytes as indicated by the expression of specific marker genes β3-tubulin (Fig. 1O), GFAP (Fig. 1P), and O4 (Fig. 1Q), respectively. Taken together, we established three NSC types R-NSCP1, R-NSCP6, and NPC, which represented early-to-late neural developmental stages and displayed decreased capacities in neural patterning and specification.

### 3.2. Gene expression signatures of the three NSC populations

**Figure 2.**
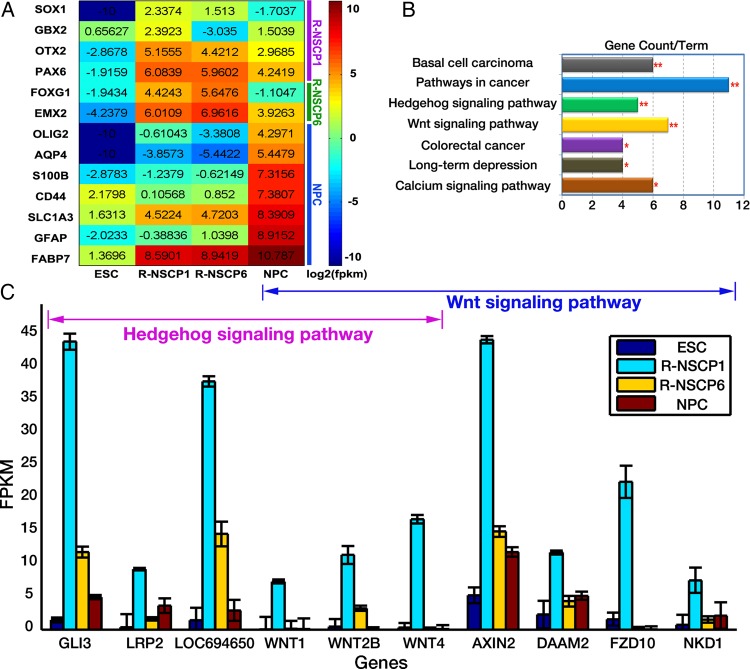
The expression patterns of prevalent genes during rESC neural differentiation. (A) Heatmap plot of expression profile of marker genes of NSCs during rESC neural differentiation. There are three groups for the marker genes, including R-NSCP1-prevalent (purple), R-NSCP6-prevalent (green), and NPC-prevalent (blue). (B) Significantly enriched GO terms associated with prevalent genes of R-NSCP1 stage. Marks * and ** represent the terms satisfy FDR <0.01 in Fisher's exact test with FDR adjustment.^33^ (C) Gene expression patterns of the Hedgehog and Wnt signalling pathway in rESC neural differentiation. The red arrow includes differentially expressed genes in the Hedgehog pathway, while the blue arrow represents the differentially expressed genes in the Wnt signalling pathway.

To investigate the regulation of monkey ESC neural differentiation and to identify the unique molecular properties of R-NSCP1, we performed parallel genome-wide analysis of mRNA and miRNA expression profiling in four samples (Supplementary Fig. S1). Respective 72, 78, 78, and 100 million sequence reads (36, 39, 39, and 50 million mate pairs) were obtained in ESCs, R-NSCP1, R-NSCP6, and NPC. Using stringent criteria FPKM ≥3 to define an ‘expressed’ gene, we detected the expression of 12,457 of 20,106 genes (∼61.96%) surveyed in at least one stage and constructed the high-resolution transcriptomes of the four samples (Supplementary Table S3). Based on these expression data, we re-examined the differentiation stage of these NSC types by looking closely at the expression patterns of the following marker genes: LOC708561 (similar to SOX1), PAX6, LOC694175 (similar to OTX2), LOC716287 (FOXG1-like), GBX2, EMX2, FABP7, SLC1A3, GFAP, S100B, AQP4, OLIG2, and CD44. SOX1 is one of the known earliest neural precursor markers and plays essential roles in neural determination. Its expression can be detected as early as in primitive neuroepithelial progenitors (NEPs), but disappears in radial glias (RG).^37,38^ PAX6 is a broad NSC marker with expression initiated in primitive NEP and sustained until the late NSCs.^38^ OTX2, FOXG1, EMX2, and GBX2 are markers of anterior identity and play roles in early establishment of anterior/posterior patterning of neural plate (e.g. GBX2) or in the regional patterning of the forebrain, midbrain, and hindbrain.^3,39–41^ Because the NSCs of early stage exhibit anterior identity, the co-expression of anterior markers with early neural markers is commonly used to mark the early NSCs with broad neural differentiation competence. FABP7, SLC1A3, GFAP, S100B, AQP4, OLIG2, and CD44 are highly transcribed in the late stage of NSC/NPC.^3^ As shown in Fig. 2A, among the six early NSC markers, PAX6, OTX2, FOXG1, and EMX2 were prevalently expressed in both R-NSCP1 and R-NSCP6 cells, whereas SOX1 and GBX2 were specifically expressed in R-NSCP1. Late-stage NSC markers FABP7 and SLC1A3 displayed prevalent expression in NPC other than in R-NSCP1 and R-NSCP6. Genes involved in the neuronal or astrocytic differentiation process (GFAP, S100B, AQP4, OLIG2, and CD44) were restrictively expressed in NPC. Moreover, hierarchical clustering analysis of these four samples revealed that R-NSCP6 was clustered with NPC instead of R-NSCP1 (Supplementary Fig. S2). Taken together, these data further supported that the regional specification and neural differentiation potentials decreased from R-NSCP1 to NPC.

We next went on to identify the unique molecular signatures of the three NSC populations. To this end, the expression fold change between the highest FPKM value and the secondary highest FPKM value was calculated for each gene. Genes with the fold changes of ≥2 were considered as stage specifically over-expressed genes. As a result, we identified 1,147, 258, 949, and 821 genes showing prevalent expression in ESC, R-NSCP1, R-NSCP6, and NPC, respectively (Supplementary Table S4). Among the 1,147 genes over-represented in ESCs, 391 were exclusively detected in ESCs (an FPKM value of <3 in other stages). The well-known conserved markers of undifferentiated ESCs including POU5f1, NANOG, DNMT3B, L1TD1, ZFP42, SALL4, LEFTY1, and PRDM14 were included in the list. LOC719865 (similar to RNA-binding protein Lin-28A) was also highly expressed in ESCs. Supplementary Table S5 summarizes the most enriched genes (fold change ≥50) with a high expression level (FPKM ≥20) in rESCs. It is interesting to note that PKM2, which encodes pyruvate kinase isoform M2 important for aerobic glycolysis, was exclusively and abundantly expressed in ESCs, suggesting that the PSCs might have distinct metabolic property from proliferating somatic stem cells.

Among the 258 R-NSCP1-prevalent genes (Supplementary Table S4), 124 were restrictively expressed in R-NSCP1 (an FPKM value of <3 in other stages). Of which, the uncharacterized genes LOC100428562 (cytochrome c oxidase subunit 2-like), LOC100428486, LOC100428944, and LOC100430630 showed a high expression level (FPKM ≥48) in the R-NSCP1 stage. Whether they play critical roles in R-NSCP1 function deserved exploration. Table 1 summarizes the typical R-NSCP1-enriched genes with relative high expression abundance (FPKM ≥10 and fold change ≥4). The expression pattern of PAX3, Col3A1, and WNT1 was randomly selected for validation by qRT–PCR (Supplementary Fig. S3A). We observed that many of the R-NSCP1-prevalent genes are well-known gene signatures in neural regionalization and patterning during neurogenesis (e.g. PAX3, PAX8, OLIG3, LGI1, GLI3, EN2, DCX, DMRTA1, and SLIT2).^42–46^ The GNF database shows that four of the nine gene signatures (including LGI1, GLI3, EN2, and DCX) are predominantly expressed in neural tissues (the expression levels in neural tissues, such as whole brain, retina, and hypothalamus, are significantly higher than the other tissues; Wilcoxon signed-rank test, *P* < 0.01). More interestingly, the DCX gene (doublecortin) is specifically expressed in the fetal brain. In addition, we annotated all the R-NSCP1-prevalent genes using human gene signatures in MSigDB^34^ (v4.0) and found that the genes up-regulated in neurons (Term ID: CAHOY_NEURONAL) were enriched (FDR <1 × 10^−10^). The Kyoto Encyclopedia of Genes and Genomes (KEGG) pathway analysis on these 258 R-NSCP1-prevalent genes revealed that these genes were highly enriched in the basal cell carcinoma, pathways in cancer, Hedgehog signalling pathway, and Wnt signalling pathway (Fisher's exact test, FDR <0.01; Fig. 2B). Six components of the Hedgehog signalling pathway including GLI3, LRP2, LOC694650, WNT1, WNT4, and WNT2B were significantly over-represented in R-NSCP1 (Fig. 2C) (Fisher's exact test, FDR <0.01). Similarly, components of the Wnt signalling pathway, including AXIN2, DAAM2, FZD10, NKD1, and the three WNT genes mentioned above, were over-represented in R-NSCP1 (Fig. 2C). The function of Hedgehog signalling in facilitating R-NSC expansion *in vitro* has been validated in the previous study.^9^ AXIN2 is a universal read-out of Wnt signalling activation, and its prevalent expression in R-NSCP1 (Supplementary Fig. S3B) indicated that Wnt signalling pathway was highly activated in R-NSCP1 cells and might play important roles in regulating the biology of early stage R-NSC. In addition, we explored the expression patterns of gene signatures in Hedgehog and Wnt signalling pathways in human tissues using the GNF database.^35,36^ It shows that 5 of the 10 gene signatures are predominantly expressed in neural tissues, including GLI3, WNT1, WNT2B, DAAM2, and FZD10 (Supplementary Fig. S4).

**Table 1.** Top list of r-NSCP1-prevalent genes during rESC neural differentiation

Gene	ESC	R-NSCP1	R-NSCP6	NPC	Gene descriptions
LOC100428562	0.00	132.67	0.00	0.00	Cytochrome c oxidase subunit 2-like
PAX3	0.06	13.54	0.01	0.03	Paired box 3
WNT4	0.28	16.80	0.04	0.08	Wingless-type MMTV integration site family, member 4
ALOX15	0.07	11.22	0.33	0.01	Arachidonate 15-lipoxygenase
COLEC12	3.60	52.53	2.73	0.02	Collectin subfamily member 12
FZD10	1.56	22.53	0.02	0.01	Frizzled family receptor 10
SDK2	0.39	16.81	1.73	0.79	Sidekick cell adhesion molecule 2
CLDN1	0.83	32.17	3.68	0.08	Claudin 1
SPOCK1	2.23	44.48	5.55	1.39	Sparc/osteonectin, cwcv and kazal-like domains proteoglycan (testican) 1
LOC713704	0.00	91.95	10.11	12.44	PR domain zinc finger protein 16-like
COL3A1	0.12	10.06	0.59	1.52	Collagen, Type III, alpha 1
RHOU	2.10	25.74	3.90	0.40	Ras homologue family member U
RGS5	2.41	23.25	3.57	0.14	Regulator of G-protein signalling 5
ZBTB16	0.16	10.93	1.76	0.32	Zinc finger and BTB domain containing 16
CBLN1	18.67	113.84	11.65	0.02	Cerebellin 1 precursor
CNTNAP2	1.87	23.47	3.86	0.11	Contactin-associated protein-like 2
PRTG	1.60	19.99	3.38	1.07	Protogenin
GNRH1	1.15	11.34	1.85	1.97	Gonadotropin-releasing hormone 1
LGI1	0.28	26.61	4.63	2.49	Leucine-rich, glioma inactivated 1
FLRT2	1.61	16.33	2.88	1.29	Fibronectin leucine-rich transmembrane protein 2
COL4A6	3.39	60.33	10.65	1.35	Collagen, Type IV, alpha 6
CA2	5.53	33.28	6.44	1.05	Carbonic anhydrase II
FAM84A	1.69	12.24	1.49	2.38	Family with sequence similarity 84, member A
TNFRSF19	2.01	22.39	2.44	5.10	Tumour necrosis factor receptor superfamily member 19-like
BMF	4.05	16.31	3.17	1.51	Bcl2 modifying factor

Compared with R-NSCP1 cells, R-NSCP6 and NPC cells displayed decreased plasticity in neural patterning and specification. In consistence with this tendency, many important transcription factors involved in CNS regionalization and specification were identified as R-NSCP6 prevalent (Table 2). These genes include TBR1, NEUROD6, NEUROD2, NEUROD1, NEUROG1, CTXN1, NR2F1, NR2F2, DMRT3, RSPO2, EMX1, BHLHE22, VSTM2L, LEAP2, RAB3a, Hes6, LY6H, MAPK11, GLI1, HMP19, FBXW9, FBXW5, DLK1, and LHX2. Apart from these known factors, many uncharacterized genes were uncovered to be highly enriched in R-NSCP6 (Supplementary Table S4). Investigating the functions of these novel genes would provide new insights into the regulation of CNS specification in primates.

**Table 2.** Top list of r-NSCP6-prevalent genes during rhesus embryonic stem cell neural differentiation

Gene	ESC	R-NSCP1	R-NSCP6	NPC	Gene descriptions
TBR1	0.17	8.16	41.92	0.071	T-box, brain, 1
NEUROD6	0	1.17	32.03	0.12	Neuronal differentiation 6
NEUROD2	0	0.06	7.11	0	Neuronal differentiation 2
NEUROG1	0	2.74	9.48	0.15	Neurogenin 1
CTXN1	4,577	4,218	120,988	1,949	Cortexin 1
NR2F1	0.12	61.90	238.9	34.81	Nuclear receptor subfamily 2, group F, member 1
NR2F2	0.37	39.99	141.06	16.45	Nuclear receptor subfamily 2, group F, member 2
DMRT3	0.02	13.7	46.30	0	Doublesex and mab-3-related transcription factor 3
RSPO2	0.3	119.7	289.86	0.83	R-spondin 2
EMX1	0	4.5	29.39	0	Empty spiracles homeobox 1
BHLHE22	0.06	0.75	10.09	0.23	Basic helix-loop-helix family, member e22
VSTM2L	0.33	1.73	23.21	2.06	V-set and transmembrane domain-containing 2 like
LEAP2	0	9.68	82.38	0	Liver-expressed antimicrobial peptide 2
RAB3A	0.60	4.63	25.26	3.0	RAB3A, member RAS oncogene family
HES6	1.23	25.9	281.62	52.88	Hairy and enhancer of split 6 (Drosophila)
LY6H	0.29	23.31	168.39	35.06	Lymphocyte antigen 6 complex, locus H
MAPK11	2.11	9.99	47.44	8.799	Mitogen-activated protein kinase 11
GLI1	1.60	2.42	11.35	1.82	GLI family zinc finger 1
HMP19 (NSG2)	0.14	20.47	83.49	1.21	Neuron-specific protein family member 2
FBXW9	3.45	5.99	22.02	6.39	F-box and WD repeat domain containing 9
FBXW5	11.85	14.9	44.68	15.8	F-box and WD repeat domain containing 5
DLK1	1.135	26.60	58.36	0.96	Delta-like 1 homologue (Drosophila)
LHX2	0.34	38.4	109.7	21.4	LIM homeobox 2

### 3.3. AS in rESC neural differentiation

**Figure 3.**
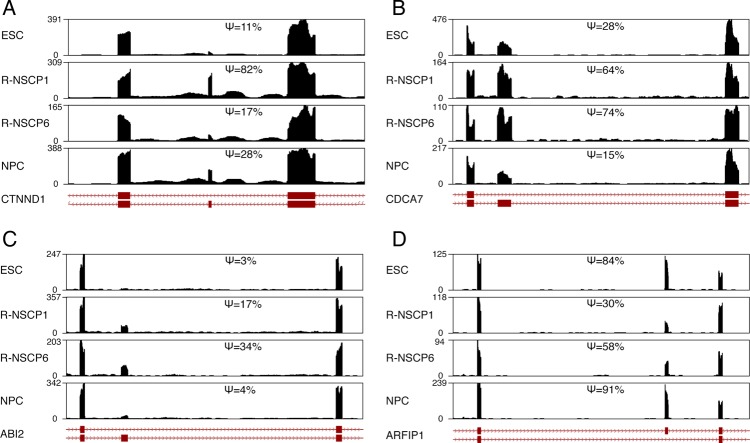
Examples of R-NSC-specific AS changes during rhesus ESC neural differentiation. The RNA-Seq reads coverage of alternative exons and the flanking exons in CTNND1, CDCA7, ABI2, and ARFIP1 are shown in A–D, respectively. The *y*-axes represent read coverage; the inclusion level (*Ψ*) of each alternative exon on each stage is shown. Three-exon isoform structures are shown at the bottom of each panel, and the middle exon is the alternative exon.

AS is an important layer of gene expression regulation and plays necessary roles in various aspects of developmental processes. To find out whether splicing switch of genes participates in the regulation of unique R-NSC properties, we used MATS^30^ to analyse AS changes during rESC neural differentiation. We focused on the exon skipping AS and identified 161 stage-specific events in all four stages (Supplementary Table S6). Among them, 28 events were R-NSC-specific. For examples, an alternative exon in CTNND1 is specifically highly included in R-NSCP1 (Fig. 3A), an alternative exon in CDCA7 shows a high inclusion level only in R-NSCs at P1 and P6 (Fig. 3B), an alternative exon in Abl-interactor 2 (ABI2) shows nearly exclusive inclusion in R-NSCs (Fig. 3C), and an alternative exon in ARFIP1 shows R-NSC-specific low inclusion (Fig. 3D). AS could result in different protein products; all the four alternative exons shown in Fig. 3 are in coding regions and inclusion of these exons results in inclusion of a certain number of amino acids encoded by the alternative exons in the protein products. For instance, the inclusion of the 183-bp alternative exon in ABI2, an SH3-domain protein involved in neuronal development, lead to protein product with additional 61 amino acids. On the other hand, AS could have other effects such as premature termination due to read frame shift. One hundred and fifty-one of the 161 stage-specific alternative exons are in protein-coding regions, even though most of them (100 of 151) cause in frame change of protein, there are 31 splice variants cause reading frame shift and 10 splice variants result in different usage of start codons (Supplementary Table S6). The R-NSC stage-specific inclusion of alternative exon suggests that the exon inclusion may be important to regulate R-NSC-specific functional properties.

### 3.4. miRNA biomarkers of NSCs and potential mRNA–miRNA regulatory interaction

miRNA expression profiling was examined by single-end small RNA sequencing. Total 32.5, 39, 33, and 36 million sequence reads were obtained from the ESC, R-NSCP1, R-NSCP6, and NPC, respectively. After mapping to the rhesus monkey miRBASE database, 451 of the 466 annotated rhesus miRNAs (∼97%) were detected with absolute reads of ≥1 in our study.

To evaluate our miRNA sequencing quality, we compared the miRNA expression profile of ESCs with that reported in Sun *et al.*'s study.^20^ By Solexa sequencing, Sun *et al.* discovered total 352 annotated miRNAs in three lines of rESCs (including IVF3.2). Here, we detected 451 miRNAs solely in ESCs from line IVF3.2. Apart from the 329 overlapping miRNAs in these two independent works, we detected additional 122 annotated miRNAs in rESCs. The full list of miRNA species was shown in Supplementary Table S7.

To find out the miRNAs prevalently expressed in each stage, the expression fold change between the highest reads count and the secondary highest reads count was calculated for each miRNA. miRNAs with the expression fold changes of ≥2 were considered as stage specifically over-represented. As a result, we identified 74, 59, 73, and 6 annotated miRNAs showing prevalent expression in ESC, R-NSCP1, R-NSCP6, and NPC, respectively (Supplementary Table S8). Among the 74 ESC-prevalent miRNAs, 11 miRNAs were restrictively expressed in monkey ESCs. The well-known ESC-enriched miRNAs including miR-302a, miR-302b, miR-302c, miR-302d, miR-367, miR-371-5p, miR-371-3p, miR-372, and miR-373 were in our list, indicating the liability of our data. We then focused on those miRNAs with high expression in R-NSCP1 or R-NSCP6. Table 3 presents the top miRNAs with the reads count of >1.0 × 10^4^ and fold change of >3.0 in R-NSCP1 or R-NSCP6.

**Table 3.** Highly expressed miRNAs in r-NSCP1 and r-NSCP6 in rhesus monkey

miRNAs	ESC	R-NSCP1	R-NSCP6	NPC	Mature_sequences
R-NSCP1 prevalent					
miR-99b	212,869	2,252,754	566,306	102,551	CACCCGTAGAACCGACCTTGCG
miR-146b-5p	22,717	247,013	61,668	10,987	TGAGAACTGAATTCCATAGGCT
miR-135a	2,711	137,160.5	33,916	8,194	TATGGCTTTTTATTCCTATGTGA
miR-20b	24,368	107,856	21,182	658	CAAAGTGCTCATAGTGCAGGTAG
miR-106a	17,754	58,830	13,913	438	AAAAGTGCTTACAGTGCAGGTAGC
miR-18b	8,136	29,118	6,400	108	TAAGGTGCATCTAGTGCAGTTAG
miR-874	4,928	15,527	4,540	717	CTGCCCTGGCCCGAGGGACCGA
miR-374a	2,796	12,882	3,576	1,500	TTATAATACAACCTGATAAGTG
R-NSCP6 prevalent					
miR-149	5,779	44,126	154,996	17,501	TCTGGCTCCGTGTCTTCACTCCC
miR-410	9,507	15,214	55,897	74	AATATAACACAGATGGCCTGT
miR-654-3p	2,936	15,011	49,798	48	TATGTCTGCTGACCATCACCTT
let-7e	1,908	16,231	48,955	7,494	TGAGGTAGGAGGTTGTATAGTT
miR-409-3p	4,325	7,020	38,577	55	GAATGTTGCTCGGTGAACCCCT
miR-381	5,215	5,655	28,323	21	TATACAAGGGCAAGCTCTCTGT
miR-889	741	4,268	15,327	18	TTAATATCGGACAACCATTGT
miR-758	988	2,422	10,903	10	TTTGTGACCTGGTCCACTACCC

**Figure 4.**
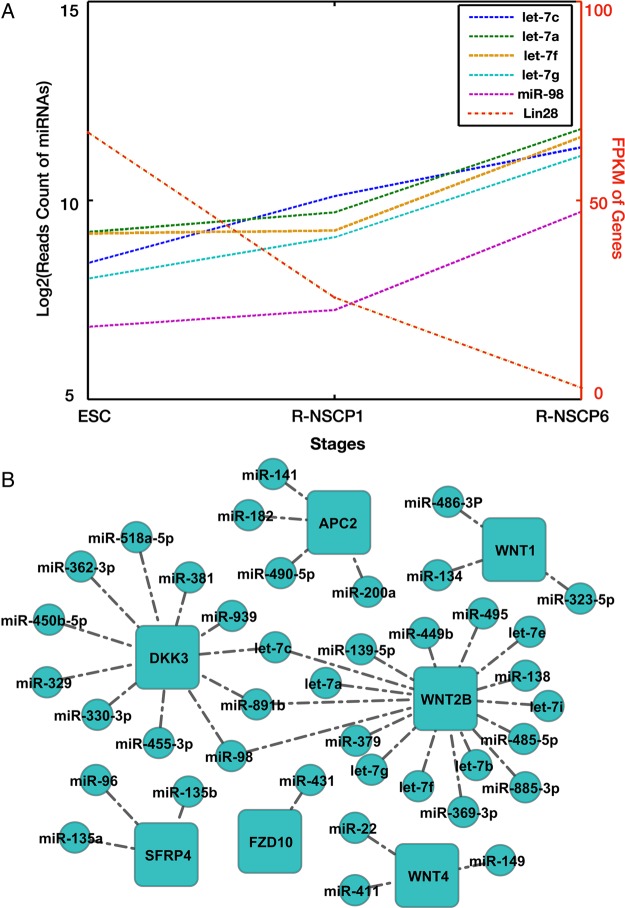
miRNAs and their target genes. (A) Expression patterns of Lin28 and its regulating miRNAs. (B) Wnt signalling pathway and potential miRNAs regulators.

miRNAs regulate gene expression at the post-transcriptional level. We then examined the possible regulatory interactions among these stage-prevalent miRNAs, mRNAs, and signalling pathways. We first looked at the expression dynamics of well-known Lin28-let7 regulatory axis. RNA-binding protein Lin28 represses the maturation of pre-let7 precursor. Down-regulation of Lin28 mRNA is accompanied with up-regulation of let-7 miRNA family, and this regulatory axis dynamics is required for ESC differentiation as well as commitment to neural fate.^47^ Indeed, our expression data clearly showed the gradual decrease in Lin28B expression from ESCs to R-NSCP6. Inversely, the expression levels of most Let-7 family members (let-7a, 7c, 7e, 7f, 7g, and miR-98) increase gradually from ESC to R-NSCP6 (Fig. 4A).

As mentioned above, Hh and Wnt signalling pathways were highly activated in R-NSCP1 but suppressed in R-NSCP6, we then asked whether there were R-NSCP1- or R-NSCP6-prevalent miRNAs implicated in regulating the pathways. The *in vivo* activities of Hh and Wnt are tightly controlled at multiple levels by variable positive as well as negative regulators. The important regulators of Hh signalling include Gas1, Ptch1, Sufu, and Gli3.^48,49^ Sufu and Ptch1 maintained stable mRNA expression during the six passages of R-NSCs, whereas Gas1 and Gli3 significantly down-regulated their expression when R-NSCs were passed into the sixth generation. We thus focused on Gas1 and Gli3 to search for their potential regulating miRNAs. To do this, we set the following criteria that the candidate miRNAs should pass through the combinatorial miRNA-target predictions (see Section 2) and display inverse expression pattern during the passage of R-NSCs. Similarly, we examined the expression dynamics of Wnt pathway components during the process of R-NSC passage. Several components of the Wnt pathway displayed drastic expression change between R-NSCP1 and R-NSCP6. These include ligands WNT1, WNT2B, WNT4, WNT8B, receptor FZD10, and three Wnt antagonists DKK3, SFRP4, and APC2. Of note, APC2 is a CNS-specific negative regulator of Wnt signalling.^50^ We searched for their potential regulating miRNAs. Figure 4B summarizes the predicted miRNAs having potential in modulating Wnt signalling intensity in R-NSCs. In the Hh signalling pathway, we detected mir-495 and mir-383 for Gas1 and mir-654-5P for Gli3.

## 4. Discussion

R-NSCs represent an early stage of neural development. Their broad neural differentiation and regional specification potentials made them attractive in cell replacement therapy. However, little is known regarding the mechanisms regulating their full neural differentiation potential. Studying the underlying molecular regulators would shed lights on their potential utilization in regenerative medicine. To our knowledge, few works had been done to systematically investigate the molecular properties of R-NSCs. By microarray analysis, Elkabetz *et al.*^9^ compared the gene expression profile of R-NSCP1 with that of NPC and identify a list of R-NSCP1-specific genes. However, due to the discrete neural differentiation stage and wide gap between R-NSCP1 and NPC, the genes highly expressed in R-NSCP1 may not be *bona fide* R-NSCP1-specific. To overcome this shortcoming, we included R-NSC at passage 6 (R-NSCP6) along with R-NSCP1 and NPC in this study. When compared with R-NSCP1, R-NSCP6 lost the morphological rosette feature as well as partial neural regionalization abilities and therefore served as transition between R-NSCP1 and NPC. Inclusion of R-NSCP6 in our experimental design could trim off the molecules not *bona fide* unique to R-NSCP1 and therefore increased the likelihood for identification of molecular regulators of R-NSCP1 functions. Indeed, many of the R-NSCP1-specific genes identified in Elkabetz *et al.*'s study were not included in our list.^9^ For instance, PLAGL1, DACH1, PLZF, NR2F1, ZNF312, LIX1, LMO3, DMRT3, FAM70A, EVI1, MMRN1, RSPO3, EMX2OS, and LEF1 were top 14 R-NSCP1-specific genes in Elkabetz's work.^9^ However, only PLZF was verified to be truly R-NSCP1 prevalent in this study (Supplementary Fig. S5). Others were equally (e.g. PLAGL1, DACH1, LEF1) or even increasingly (e.g. NR2F1 and DMRT3) expressed in R-NSCP6 compared with R-NSCP1, although their abundance in R-NSCP1 was significant higher than that in NPC (Supplementary Fig. S5). Thus, our ESC neural differentiation system provided a better platform to study the molecular properties of R-NSCs. Furthermore, we utilized high-throughput deep sequencing instead of microarray-based approaches for systemic analysis. Deep sequencing has the advantage over microarray in discovery of novel genes or miRNAs without bias and therefore can largely broaden our knowledge on the regulation of monkey ESC neural differentiation.

Rhesus monkey is considered as a better non-primate animal model than mouse in biomedical research. Comparison of our data with human PSC neural differentiation supported that the expression patterns in ESC neural differentiation are similar between human and rhesus monkeys. For example, Wu *et al.*^51^ applied high-throughput deep sequencing to analyse the transcriptome changes that occur during the differentiation of human ESCs into the neural lineage. Many common gene signatures enriched for Wnt and Hedgehog signalling pathways are shared between human and rhesus (WNT1, WNT4, NKD1, etc.) in these two studies. We also compared the most important gene signatures in mouse ESC neural differentiation^52^ with our R-NSC-prevalent genes and found that the many of the gene signatures (69 of 539 rhesus mouse ortholog pairs from rhesus R-NSCP-prevalent genes; Supplementary Table S9) are conserved in the neural differentiation process, such as COL4A6, SPOCK1, TNFRSF19, FBXW5, HES6, LHX2, NR2F1, NR2F2, and RAB3A (Tables 1 and 2). However, there exist species-specific genes. For example, Wnt3a and Wnt5b might be mouse-specific, while Wnt2b is active in rhesus monkey. These comparisons between species to derive the conserved and divergent genes/pathways will be of critical importance in building suitable animal models for human diseases.

In summary, by whole-genome comparison of mRNA and miRNA expression profiles, we identified a set of mRNAs, miRNAs, and signalling pathways exclusively or prevalently expressed/activated in R-NSCP1 or R-NSCP6. The possible regulatory interactions between mRNAs and miRNAs were also discussed. These molecules or signalling pathways could participate in the control of the full neural specification and differentiation potentials of R-NSCs, and their exact roles await future investigation.

## 5. Availability

The mRNA-seq and miRNA-seq data sets are available at http://www.ncbi.nlm.nih.gov/geo/query/acc.cgi?token=qpshkseilzclvwv&acc=GSE53260 and via GEO (accession GSE53260).

## Supplementary data

Supplementary data are available at www.dnaresearch.oxfordjournals.org.

## Funding

This work was supported by the National Key Basic Research Program of China (grant number 2011CBA01101).

## Supplementary Material

Supplementary Data
