# Supplementary material for: mRNA-Seq and MicroRNA-Seq Whole-Transcriptome Analyses of Rhesus Monkey Embryonic Stem Cell Neural Differentiation Revealed the Potential Regulators of Rosette Neural Stem Cells
Source: DNA Res. 2014 Jun 17;21(5):541–54. doi: 10.1093/dnares/dsu019 (PMC4195499; doi:10.1093/dnares/dsu019)
Supplement: Supplementary Data [file supp_21_5_541__index.html]

mRNA-Seq and MicroRNA-Seq Whole-Transcriptome Analyses of Rhesus Monkey Embryonic Stem Cell Neural Differentiation Revealed the Potential Regulators of Rosette Neural Stem Cells — Supplementary Data 

# mRNA-Seq and MicroRNA-Seq Whole-Transcriptome Analyses of Rhesus Monkey Embryonic Stem Cell Neural Differentiation Revealed the Potential Regulators of Rosette Neural Stem Cells

## Supplementary Data

Supplementary Data

**Files in this Data Supplement:**

- Supplementary Figure 1 - pdf file
- Supplementary Figure 2 - pdf file
- Supplementary Figure 3 - pdf file
- Supplementary Figure 4 - pdf file
- Supplementary Figure 5 - pdf file
- Supplementary Table 1and2 - doc file
- Supplementary Table 3 - xls file
- Supplementary Table 4 - xls file
- Supplementary Table 5 - xls file
- Supplementary Table 6 - xls file
- Supplementary Table 7 - xls file
- Supplementary Table 8 - xls file
- Supplementary Table 9 - xls file
