# Supplementary material for: mRNA-Seq and MicroRNA-Seq Whole-Transcriptome Analyses of Rhesus Monkey Embryonic Stem Cell Neural Differentiation Revealed the Potential Regulators of Rosette Neural Stem Cells
Source: DNA Res. 2014 Jun 17;21(5):541–54. doi: 10.1093/dnares/dsu019 (PMC4195499; doi:10.1093/dnares/dsu019)
Supplement: Supplementary Data [file supp_dsu019_dsu019supp_fig1.pdf]

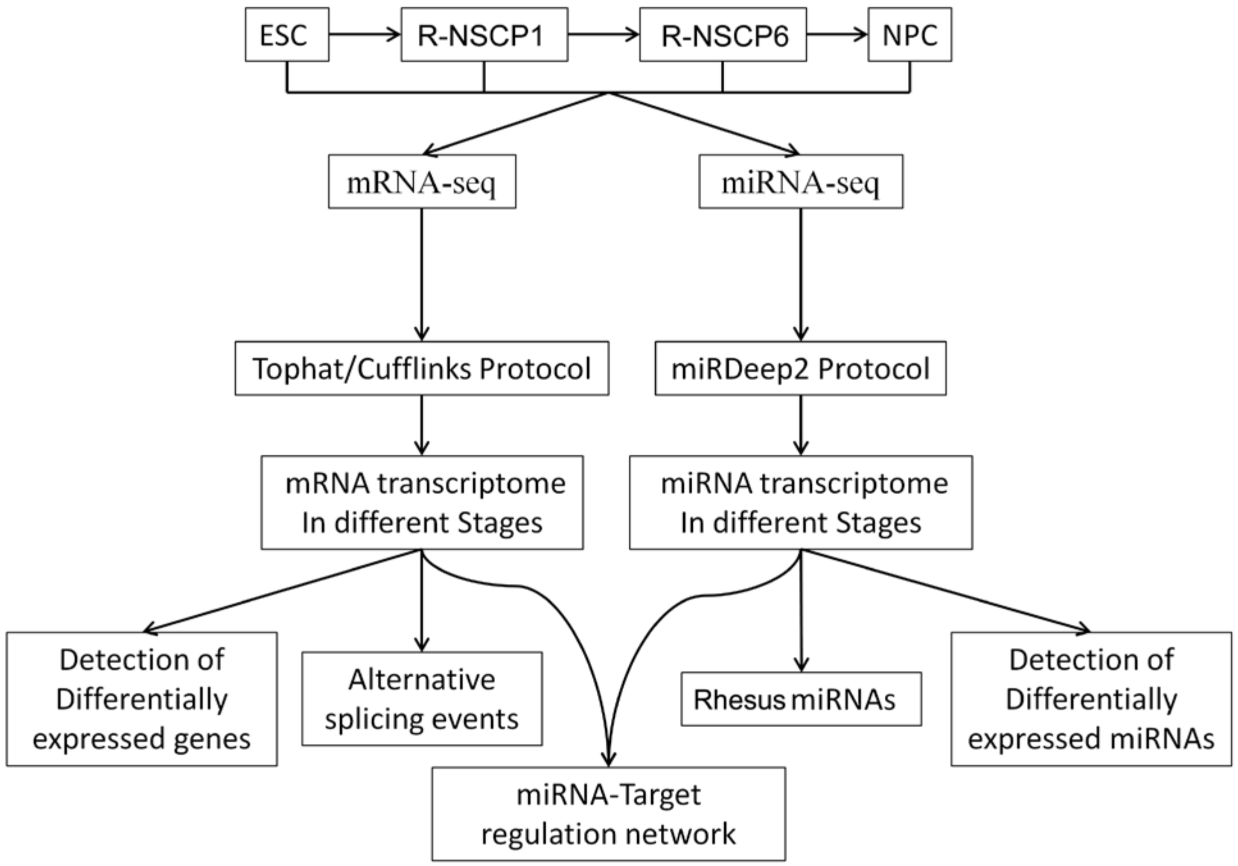

Supplementary Figure S1. Study design. We performed parallel genome-wide analysis of mRNA and miRNA expression profiling in four stages from rhesus monkey ESC neural differentiation, including ESCs, R-NSCP1, R-NSCP6, and NPC.
