# Supplementary material for: mRNA-Seq and MicroRNA-Seq Whole-Transcriptome Analyses of Rhesus Monkey Embryonic Stem Cell Neural Differentiation Revealed the Potential Regulators of Rosette Neural Stem Cells
Source: DNA Res. 2014 Jun 17;21(5):541–54. doi: 10.1093/dnares/dsu019 (PMC4195499; doi:10.1093/dnares/dsu019)
Supplement: Supplementary Data [file supp_dsu019_dsu019supp_fig3.pdf]

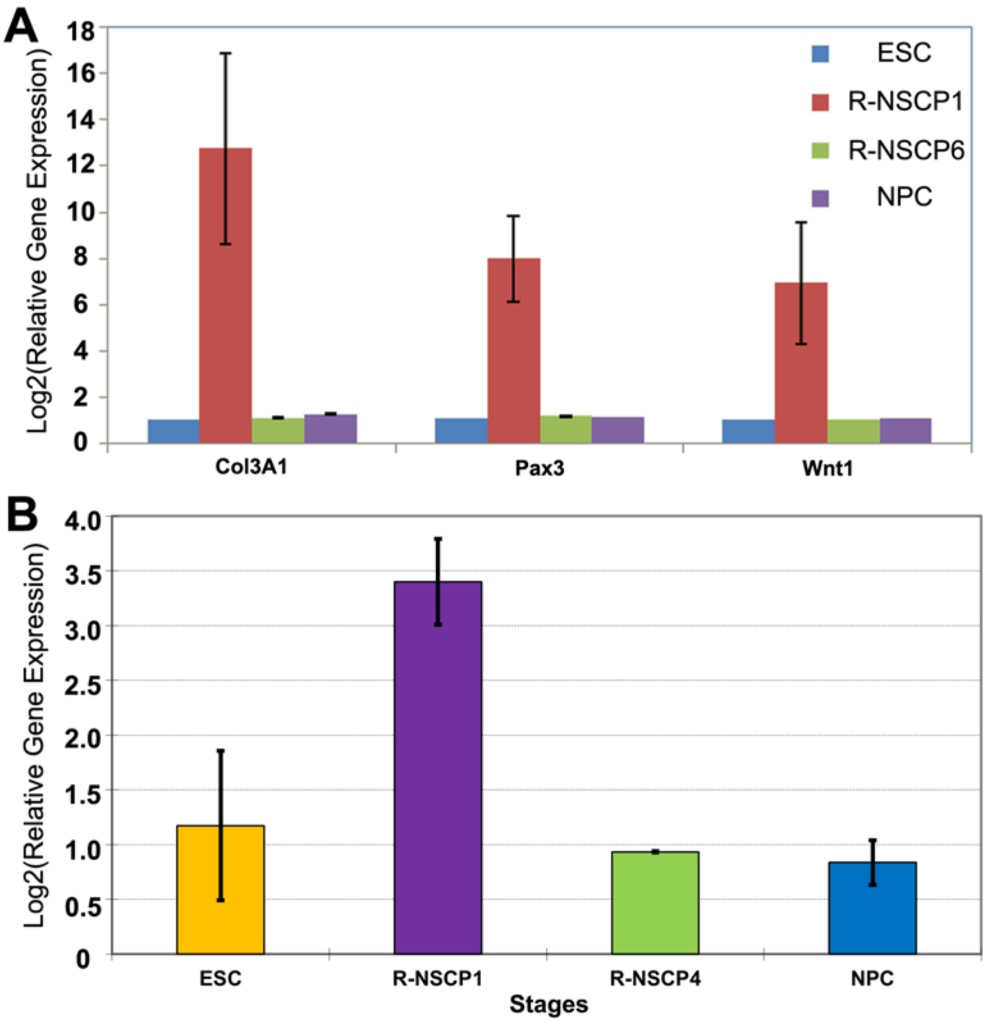

Supplementary Figure S3. Validation of signature genes using qRT-PCR. Panel (A) indicates Expression profiles for PAX3, Col3A1, and WNT1 in ESCs, R-NSCP1, R-NSCP6, and NPC. Panel (B) is the qRT-PCR results for gene AXIN2 during in ESCs, R-NSCP1, R-NSCP4, and NPC. R-NSCP4 is defined as the fourth passage of rosettes culture, which is similar to R-NSCP6.
