# Supplementary material for: mRNA-Seq and MicroRNA-Seq Whole-Transcriptome Analyses of Rhesus Monkey Embryonic Stem Cell Neural Differentiation Revealed the Potential Regulators of Rosette Neural Stem Cells
Source: DNA Res. 2014 Jun 17;21(5):541–54. doi: 10.1093/dnares/dsu019 (PMC4195499; doi:10.1093/dnares/dsu019)
Supplement: Supplementary Data [file supp_dsu019_dsu019supp_fig4.pdf]

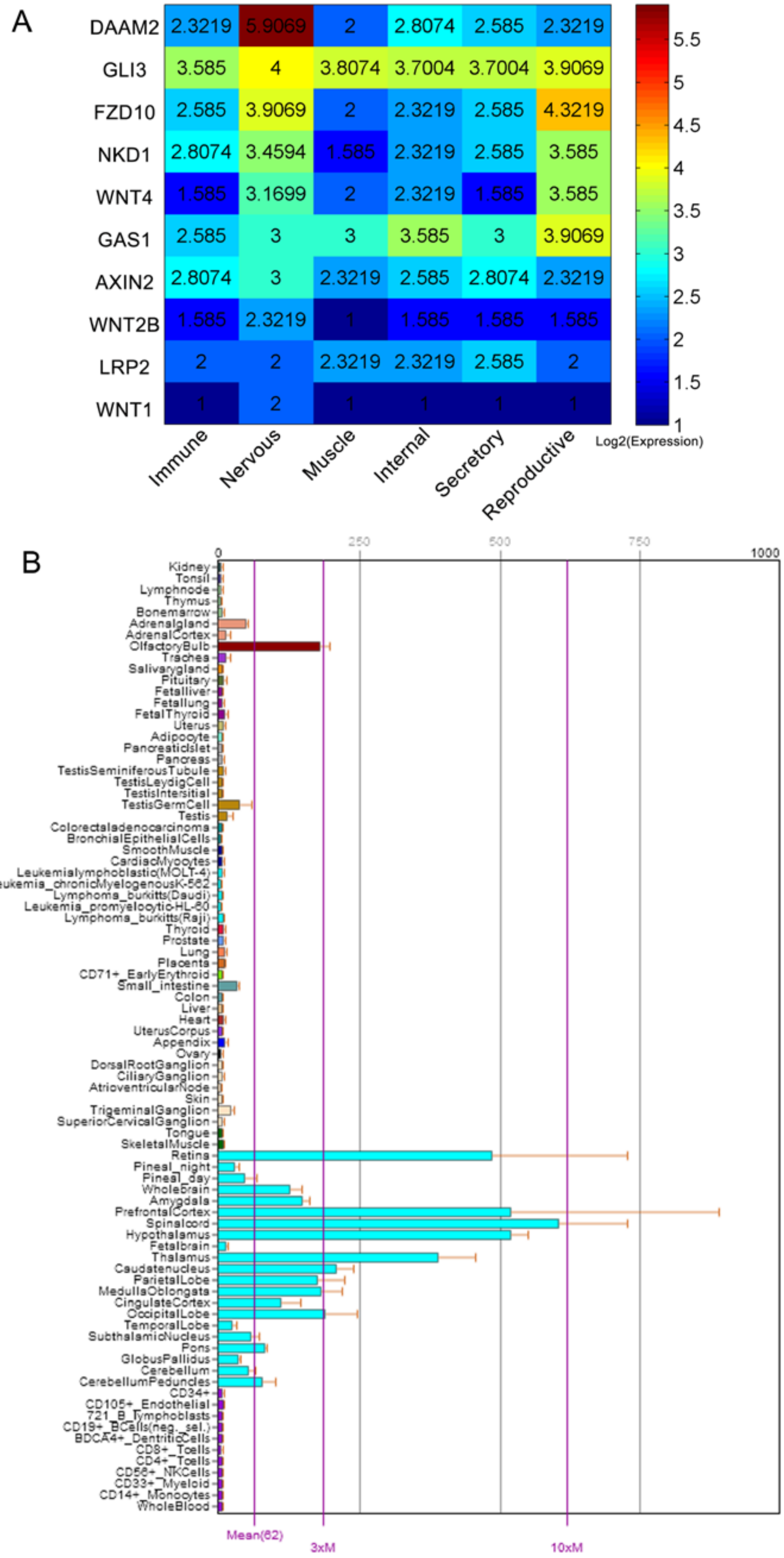

A
DAAM2
GLI3
FZD10
NKD1
WNT4
GAS1
AXIN2
WNT2B
LRP2
WNT1
2.3219 5.9069 2 2.8074 2.585 2.3219
3.585 4 3.8074 3.7004 3.7004 3.9069
2.585 3.9069 2 2.3219 2.585 4.3219
2.8074 3.4594 1.585 2.3219 2.585 3.585
1.585 3.1699 2 2.3219 1.585 3.585
2.585 3 3 3.585 3 3.9069
2.8074 3 2.3219 2.585 2.8074 2.3219
1.585 2.3219 1 1.585 1.585 1.585
2 2 2.3219 2.3219 2.585 2
1 2 1 1 1 1
Immune
Nervous
Muscle
Internal
Secretory
Reproductive
5.5
5
4.5
4
3.5
3
2.5
2
1.5
1
Log2(Expression)
B
0
250
500
750
1000
Kidney
Tonsil
Lymphnode
Thymus
Bonemarrow
Adrenalgland
AdrenalCortex
OlfactoryBulb
Trachea
Salivarygland
Pituitary
Fetalliver
Fetallung
FetalThyroid
Uterus
Adipocyte
PancreaticIslet
Pancreas
TestisSeminiferousTubule
TestisLeydigCell
TestisInterstitial
TestisGermCell
Testis
Colorectaladenocarcinoma
BronchialEpithelialCells
SmoothMuscle
CardiacMyocytes
Leukemialymphoblastic(MOLT-4)
Leukemia_chronicMyelogenousK-562
Lymphoma_burkitts(Daudi)
Leukemia_promyelocytic-HL-60
Lymphoma_burkitts(Raji)
Thyroid
Prostate
Lung
Placenta
CD71+_EarlyErythroid
Small_intestine
Colon
Liver
Heart
UterusCorpus
Appendix
Ovary
DorsalRootGanglion
CiliaryGanglion
AtrioventricularNode
Skin
TrigeminalGanglion
SuperiorCervicalGanglion
Tongue
SkeletalMuscle
Retina
Pineal_night
Pineal_day
WholeBrain
Amygdala
PrefrontalCortex
Spinalcord
Hypothalamus
Fetalbrain
Thalamus
Caudatenucleus
ParietalLobe
MedullaOblongata
CingulateCortex
OccipitalLobe
TemporalLobe
SubthalamicNucleus
Pons
GlobusPallidus
Cerebellum
CerebellumPeduncles
CD34+
CD105+_Endothelial
721_B_lymphoblasts
CD19+_BCells(neg._sel.)
BDCA4+_DentriticCells
CD8+_Tcells
CD4+_Tcells
CD56+_NKCells
CD33+_Myeloid
CD14+_Monocytes
WholeBlood
Mean(62)
3xM
10xM

Supplementary Figure S4. Expression patterns of gene signatures in Hedgehog and Wnt signaling pathways in human tissues. (A) Heatmap plot of expression levels of gene signatures involved in Hedgehog and Wnt signaling pathways in six human systems, including Immune, Nervous, Muscle, Internal, Secretory, and Reproductive organs. **(B)** Gene DAAM2 (dishevelled associated activator of morphogenesis 2) belongs Wnt signaling pathway and is an R-NSC prevalent gene signature. The figure shows that the gene is predominantly expressed in human neural tissues.
