# Supplementary material for: mRNA-Seq and MicroRNA-Seq Whole-Transcriptome Analyses of Rhesus Monkey Embryonic Stem Cell Neural Differentiation Revealed the Potential Regulators of Rosette Neural Stem Cells
Source: DNA Res. 2014 Jun 17;21(5):541–54. doi: 10.1093/dnares/dsu019 (PMC4195499; doi:10.1093/dnares/dsu019)
Supplement: Supplementary Data [file supp_dsu019_dsu019supp_fig5.pdf]

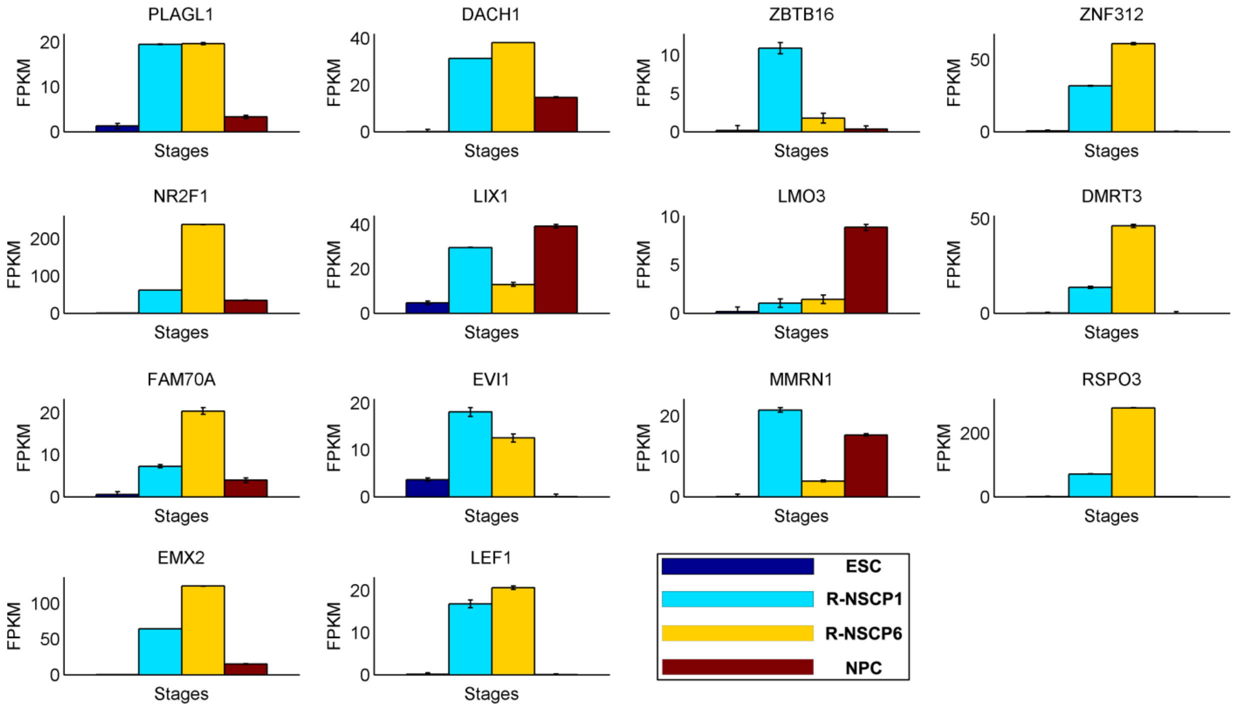

Supplementary Figure S5. Expression patterns of orthologs of human R-NSCP1 marker genes in rhesus monkey ESC neural differentiation.
