# Supplementary material for: mRNA-Seq and MicroRNA-Seq Whole-Transcriptome Analyses of Rhesus Monkey Embryonic Stem Cell Neural Differentiation Revealed the Potential Regulators of Rosette Neural Stem Cells
Source: DNA Res. 2014 Jun 17;21(5):541–54. doi: 10.1093/dnares/dsu019 (PMC4195499; doi:10.1093/dnares/dsu019)
Supplement: Supplementary Data [file supp_dsu019_dsu019supp_table1and2.doc]

**Table S1. Antibodies used in neural differentiation study.**

| Antibody | Source | Dilution |
| --- | --- | --- |
| Oct4 | SantaCruz | 1:100 |
| ZO-1 | BD-BioScience | 1:100 |
| Sox2 | Abcam | 1:100 |
| Nestin | Millipore | 1:500 |
| Dach1 | SantaCruz | 1:100 |
| PLZF | SantaCruz | 1:100 |
| Forse1 | DSHB | 1:75 |
| N-cad | Sigma | 1:100 |
| En1 | Abnova Corporation | 1:50 |
| HB9 | DSHB | 1:50 |
| β3-tublin | Abcam | 1:400 |
| Msx1 | DSHB | 1:50 |
| Nkx2.2 | DSHB | 1:100 |
| S100b | BD-BioScience | 1:1000 |
| GFAP | SantaCruz | 1:100 |
| O4 | Millipore | 1:100 |
| TR-goat-anti-mouse IgG1 | SantaCruz | 1:200 |
| FITC-goat-anti-mouse IgG | SantaCruz | 1:200 |
| FITC-goat-anti-mouse IgM | SantaCruz | 1:200 |
| TR-goat-anti-rabbit IgG | SantaCruz | 1:200 |
| FITC-goat-anti-rabbit IgG | SantaCruz | 1:200 |
| FITC-goat-anti-mouse IgG2a | SantaCruz | 1:200 |
| FITC-goat-anti-mouse IgG2b | SantaCruz | 1:200 |

**Table S2. PCR primers and condition for qRT-PCR.**

| **Genes** | **Primer sequences** | **Annealing temp** | **Products** | **cycles** |
| --- | --- | --- | --- | --- |
| **GAPDH** | 5′-AACATCATCCCTGCCTCTAC-3′ | 55℃ | 236bp | 30 |
|  | 5′-GACGCCTGCTTCACTACC-3′ |  |  |  |
| **Axin2** | 5′-ATGCGTGGATACCTTAGACTTC-3′ | 55℃ | 188bp | 35 |
|  | 5′-TCTGCTGCTTCTTGATGCC-3′ |  |  |  |
| **Pax3** | 5' GAG GCC CGA GTA CAG GT 3' | 55℃ | 125bp | 35 |
|  | 5' GTC GGC ATG GCA GTG G 3' |  |  |  |
| **Col3a1** | 5' GGC AAA GAT GGA ACC AGT 3' | 55℃ | 166bp | 35 |
|  | 5' CAG CGG CTC CAA CAC C 3' |  |  |  |
| **Wnt1** | 5' ACC TGG GGA CTC CTC AAA CC 3' | 58℃ | 150bp | 35 |
|  | 5' CCC ATC CTT CTC CCA CCT 3' |  |  |  |
